# Incorporating Climate Change and Exotic Species into Forecasts of Riparian Forest Distribution

**DOI:** 10.1371/journal.pone.0107037

**Published:** 2014-09-12

**Authors:** Dana H. Ikeda, Kevin C. Grady, Stephen M. Shuster, Thomas G. Whitham

**Affiliations:** 1 Department of Biological Science, Northern Arizona University, Flagstaff, Arizona, United States of America; 2 Merriam-Powell Center for Environmental Research, Northern Arizona University, Flagstaff, Arizona, United States of America; 3 School of Forestry, Northern Arizona University, Flagstaff, Arizona, United States of America; National University of Mongolia, Mongolia

## Abstract

We examined the impact climate change (CC) will have on the availability of climatically suitable habitat for three native and one exotic riparian species. Due to its increasing prevalence in arid regions throughout the western US, we predicted that an exotic species, *Tamarix*, would have the greatest increase in suitable habitat relative to native counterparts under CC. We used an ecological niche model to predict range shifts of *Populus fremontii*, *Salix gooddingii*, *Salix exigua* and *Tamarix*, from present day to 2080s, under five general circulation models and one climate change scenario (A1B). Four major findings emerged. 1) Contrary to our original hypothesis, *P. fremontii* is projected to have the greatest increase in suitable habitat under CC, followed closely by *Tamarix*. 2) Of the native species, *S. gooddingii* and *S. exigua* showed the greatest loss in predicted suitable habitat due to CC. 3) Nearly 80 percent of future *P. fremontii* and *Salix* habitat is predicted to be affected by either CC or *Tamarix* by the 2080s. 4) By the 2080s, 20 percent of *S. gooddingii* habitat is projected to be affected by both *Tamarix* and CC concurrently, followed by *S. exigua* (19 percent) and *P. fremontii* (13 percent). In summary, while climate change alone will negatively impact both native willow species, *Tamarix* is likely to affect a larger portion of all three native species' distributions. We discuss these and other results in the context of prioritizing restoration and conservation efforts to optimize future productivity and biodiversity. As we are accounting for only direct effects of CC and *Tamarix* on native habitat, we present a possible hierarchy of effects- from the direct to the indirect- and discuss the potential for the indirect to outweigh the direct effects. Our results highlight the need to account for simultaneous challenges in the face of CC.

## Introduction

Climate change (CC) is predicted to be one of the leading causal agents of future extinctions, impacting biodiversity and ecosystem functioning worldwide [1], [2]. There is considerable evidence that shifts in species distributions both pole-ward and along elevation clines are already occurring [3]–[7], with regional die-off of species at the trailing edges of their range [8]–[11]. In the western U.S., recent climatic conditions have become more arid [12]; resulting in high mortality of numerous foundation species at the trailing edge of their distributions, in ecosystems that span from chaparral to alpine forests [10], [13]. Increasing aridity is expected to continue under CC, with models projecting higher temperatures and an increase in drought events in the next 80 years [12], [14], [15].

Projected increases in temperature and drought will negatively impact riparian ecosystems worldwide that have already experienced extensive modifications over the last century [16]–[18]. Changes in land use, particularly the alteration of flow regimes by damming [19] and extensive cattle grazing over the last century within riparian corridors, have resulted in a 97 percent decline of pre-20^th^ century riparian habitat in the western U.S. [16]. In the southwestern U.S., riparian ecosystems are dominated by *Populus* and *Salix* species [20], which are considered to be foundation species that structure community composition across multiple trophic levels and influence ecosystem processes such as nutrient cycling [21], [22]. Because riparian tree fitness is influenced by a number of different processes (e.g., temperature, soil water availability, flooding regimes), CC will impact riparian species directly by altering growth, phenology and geographic distributions, and indirectly by altering flood regimes, such as the timing of spring runoff and the magnitude of floods (reviewed in [23]).

Rising temperatures, altered precipitation, and novel disturbances associated with land use also make these riparian ecosystems particularly vulnerable to invasive exotic plant species such as those within the *Tamarix* genus [19], [24], [25]. *Tamarix* spp. (*Tamarix chinensis*, *Tamarix ramosissima* and hybrids [26], hereafter referred to as *Tamarix*) have invaded much of the southwestern U.S., making them the third most frequently occurring woody riparian plant in the region [27]. The presence of *Tamarix* can detrimentally impact native riparian trees when combined with other stressors. In areas where the natural flood regime has been altered, such as along dammed river systems, *Tamarix* has invaded habitat once dominated by native *Populus* and *Salix* species [28], [29]. Once established, *Tamarix* has been shown to outcompete native species due to its ability to tolerate a wider range of salinity and soil moisture contents than its native counterparts [29]. In systems where *Tamarix* forms dense monocultures, *Tamarix* has been shown to alter soil salinity [30], [31], hydrology [32], [33], and change the surrounding floral and faunal communities including mycorrhizal mutualists [29], [34], [35]. As such, we also consider species within the *Tamarix* genus to be exotic foundation species [21] due to their role in redefining riparian communities.

An important step toward understanding the impact of CC on riparian ecosystems in the southwest is to assess how CC and a highly competitive species (*Tamarix*) may combine or act synergistically to further extirpate native species. We used the Maxent modeling algorithm [36] to identify areas of expansion and contraction of three native riparian species (*Populus fremontii*, *Salix exigua*, *Salix gooddingii*) and the exotic *Tamarix* under one future climate change scenario and multiple general circulation models (GCMs). Although other studies have created habitat suitability models for *Tamarix* (e.g., [37], [38]), no previous modeling efforts have identified regions where CC is projected to result in 1) native habitat loss and *Tamarix* gain, and 2) potential new range overlaps between natives and *Tamarix*.

Native riparian trees have largely been ignored in research using ecological niche models (ENMs) to assess the impact of CC, especially in respect to altered temperature and precipitation regimes. Projected increases in temperature may exceed the range of tolerance of many native riparian trees [39], [40], (e.g. record temperatures >50°C [39]) resulting in a decline of these stabilizing species, while these same conditions may promote the spread of invasive species [27]. Numerous studies have reported significant effects of temperature and drought on the productivity of riparian species. Grady *et al*. [39] found populations of the riparian trees *P. fremontii* and *S. gooddingii* from warm environments were more productive in a warm common garden than were populations that were from cooler locations, but populations of the riparian shrub *S. exigua* did not perform better based on home site temperature, suggesting local adaptation to temperature in riparian trees but not shrubs. There is conflicting evidence regarding the response of *P. fremontii* and *S. gooddingii* to drought. Some studies predict that *P. fremontii* is more susceptible to drought than *S. gooddingii* [29], [41], whereas others suggest that *S. gooddingii* is less drought-tolerant [42]. Hultine *et al*. [43] found *P. fremontii* to be more sensitive to short-term drought, whereas *S. exigua* had greater sensitivity to long-term drought events.

While it is unclear which native species will be the most susceptible to drought and temperature increases, many studies have found *Tamarix* to be particularly resilient to drought and increased temperature [29], [42], [44] relative to their native counterparts. Together, these studies indicate that climate-induced effects on species' physiological tolerances have the potential to impact their geographic distributions.

In this study we examine effects of CC and exotic species on riparian ecosystems, as quantified by shifts in the geographic distribution of suitable habitat, and test the hypothesis that there will be predictable geographic responses of riparian species to CC. Based on previous studies on the physiological tolerances of native and exotic riparian species to temperature, we predict: 1) *Tamarix* will experience the greatest habitat increase as a result of climate change, with moderate declines of *S. exigua* and extensive habitat reduction of *S. gooddingii*, and *P. fremontii*. This prediction is based on work by Grady et al. [39], which found the productivity of *P. fremontii* to be most sensitive to temperature transfer distance, followed by *S. gooddingii* and *S. exigua*. 2) A greater proportion of future native riparian habitat, relative to current, will be affected by encroachment of *Tamarix* under CC. 3) Cumulative effects of *Tamarix* invasion and habitat loss due to climate change will be most prevalent at the trailing edges of native species distributions where they are already suffering the greatest abiotic stress. By assessing the impact of CC on the distribution of both native and invasive foundation species, we can begin to address the potential combined effects of CC and invasive species on the viability of threatened riparian species distributions.

## Methods

### Species Data

We obtained *P. fremontii*, *S. exigua*, *S. gooddingii*, and *Tamarix* location points from the Global Biodiversity and Information Facility (GBIF, http://www.gbif.org). Due to the prevalence of hybrids between *T. chinensis* and *T. ramosissima* which are unique to the western United States, we combined occurrences of both species in the U.S. into one *Tamarix* layer [45]. Although there have been questions regarding the accuracy of GBIF data [46], it represents the most comprehensive online database of species location points available. We used an established process to verify locations in the database [47]. Specifically, we removed location points that did not have associated coordinates or were not based on land, and localities where the recorded county did not match with the actual county of collection. A total of 5,758 points were removed in the above process resulting in 739 points for *P. fremontii*, 893 for *S. gooddingii*, 2,092 for *S. exigua* and 1,309 for *Tamarix* that cover their geographic ranges in western North America.

### Environmental Variables

Although the species modeled here occupy riparian habitat and are dependent upon environmental variables besides climate, such as flooding, soil salinity, and distance to water [48]–[50], [37], the focus of this study was to identify areas of climate expansion and contraction across a broad geographic range which could be used to prioritize conservation and restoration efforts. Species within the *Populus* and *Salix* genera are largely dependent upon dynamic river flows and occasional flooding for natural recruitment and survival [51]. However, climate variables typically explain more of the distribution across a large spatial extent than other environmental variables such as soil type and biotic interactions [52]. As such, by including bioclimatic variables to characterize the distribution of these riparian foundation species, we quantify the climatic niche of where *P. fremontii*, *S. exigua* and *S. gooddingii* could occur if flow regimes were suitable.

We used bioclimatic data from the WorldClim database [53] to project the current and future distributions of suitable habitat of all species. To reduce the number of variables included in the final model, we did a preliminary model run for each species and used Maxent's jackknife estimate to examine the permutation importance of the 19 bioclimatic variables (Table 1). Variables with contribution scores <5% were removed from the final model [54]. Variables were averaged for the time periods 1961–1990 and were at a 30 arc-second (∼1 km) resolution.

**Table 1:** The percent contribution and permutation importance (in bold) each bioclimatic variable made to the model building process.

	Species			
Variable	*P. fremontii*	*S. exigua*	*S. gooddingii*	*Tamarix*
BIO1 (Annual Mean Temperature)	**28.98**	**29.36**	**-**	**-**
	18.91	17.66		
BIO3 (Isothermality (BIO2/BIO7))	**-**	**-**	**18.87**	**10.04**
			17.68	19.98
BIO4 (Temperature Seasonality (Standard Deviation))	**23.80**	**46.39**	**15.83**	**28.91**
	36.71	61.12	5.96	23.22
BIO5 (Max Temperature of Warmest Month)	**16.17**	**-**	**4.52**	**-**
	13.47		9.67	
BIO6 (Min Temperature of Coldest Month)	**18.13**	**-**	**-**	**6.64**
	12.12			1.34
BIO7 (Temperature Annual Range (BIO5-BIO6))	**-**	**-**	**12.86**	**-**
			6.22	
BIO10 (Mean Temperature of Warmest Quarter)	**-**	**-**	**-**	**13.62**
				18.05
BIO11 (Mean Temperature of Coldest Quarter)	**-**	**-**	**-**	**17.97**
				2.58
BIO12 (Annual Precipitation)	**5.64**	**3.75**	**9.29**	**17.67**
	7.83	2.67	17.67	31.43
BIO13 (Precipitation of Wettest Month)	**-**	**-**	**9.19**	**-**
			4.32	
BIO14 (Precipitation of Driest Month)	**-**	**-**	**13.17**	**-**
			11.86	
BIO15 (Precipitation Seasonality (Coefficient of Variation))	**7.28**	**5.43**	**16.26**	**5.15**
	10.96	2.32	26.62	3.39
BIO17 (Precipitation of Driest Quarter)	**-**	**15.07**	**-**	**-**
		16.23		
Total Temperature	**87.08**	**75.75**	**52.09**	**77.18**
	81.21	78.78	39.54	65.18
Total Precipitation	**12.92**	**24.25**	**47.91**	**22.82**
	18.79	21.22	60.46	34.82

We used a conservative, moderate growth carbon emissions scenario utilized by many scientists [55], A1B [56], and two future time frames, 2050s and 2080s (averaged from 2040 to 2069 and 2070 to 2099, respectively). Because uncertainty in forecasting future climates is mainly attributed to differences in GCMs [57], we used five GCMs that best reflect the current climate of the southwestern US [58]: National Center for Atmospheric Research (NCAR) Community Climate System Model, version 3.0 (CCSM3), Max Planck Institute for Meteorology, Germany (ECHAM5/MPI), Commonwealth Scientific and Industrial Research Organisation CSIRO, Australia (CSIRO-MK3), Centre National de Recherches Meteorologiques, Meteo France, France (CNRM-CM3), and Hadley Centre for Climate Prediction and Research, Met Office United Kingdom, and Hadley Centre Global Environmental Model, version 1 (HadGEM1). These models were statistically downscaled to a 30 arc-second resolution using the Delta Method [59] and downloaded from Global Climate Model data portal (http://www.ccafs-climate.org/data/).

### Model Calibration & Evaluation

We used Maxent version 3.3.3e (http://www.cs.princeton.edu/~schapire/maxent) to forecast potential future shifts in suitable habitat of the four species in response to CC. Maximum entropy (Maxent) is a machine learning algorithm which estimates a species ecological niche by finding the distribution with the maximum entropy subject to constraints by environmental variables chosen *a priori* [36], [60]. Studies have shown Maxent to perform well as compared with other presence-background algorithms [49], [61], [62]. Since the selection of background points in Maxent can influence model performance [63], [64], we restricted the selection of background points to the sampling extent using a Minimum Convex Polygon [65]. Additionally, since the species occurrence data did not come from a random sample, there is the potential for sampling bias in the datasets. To remove potential biases, we followed the methods of Elith et al. [66] to create a Kernel Density Estimator (KDE) surface, rescaled from one to 20, from which to draw the 10,000 random background points. To avoid projecting into environments outside those which the models were trained upon, we specified the ‘fade-by-clamping’ option in Maxent, which removed heavily clamped pixels from the final model predictions [36].

Output format was specified to a logistic form for easy interpretation. We used a 10-fold cross-validation method, which holds 10% of the data for testing and trains the model on the remaining 90% for 10 iterations, in order to test the predictive performance on withheld data [67]. Model performance was evaluated using partial receiver operator characteristics (pROC) following the methods described in Peterson et al. [68]. We utilized the partial ROC software developed by N. Barve [69] in which we specified 1000 iterations with the omission threshold set at ten percent [70]. T-tests were performed to test for statistical significance of pROC values. Linear, quadratic, product, hinge and threshold functions of predictor variables were employed and variable importance was assessed using a jackknife analysis [36]. A binomial probability test was used to assess the accuracy of each predicted distribution compared to that expected by chance [36]. We evaluated variable importance by examining the permutation importance each bioclimatic variable had on the model building process.

To compare suitable and unsuitable habitats, we specified a 10th percentile training presence threshold (T10), which is a fixed threshold where only the lowest 10 percent of predicted values are rejected [70].

### Post-hoc Analyses and Calculation of Species Distribution Overlaps

We performed a number of transformations and analyses on the projected habitat models. First, we used “Raster Calculator” in ArcGIS Desktop 9.3 © [71] to identify areas of habitat loss and expansion by subtracting future habitat layers from the previous time period once a threshold had been applied. Second, to identify areas where *Tamarix* would likely interact with *P. fremontii*, *S. exigua* and *S. gooddingii*, we calculated the projected future overlap between *Tamarix* and the three native species once a threshold had been applied, using “Raster Calculator”. Regions where native habitat loss due to CC and future *Tamarix* overlapped were classified as areas where *Tamarix* would have the highest potential to displace *P. fremontii, S. exigua* and *S. gooddingii*. To minimize uncertainty pertaining to differences in GCM projections, all calculations using future projected distributions were based on a liberal threshold (T10) agreed upon by four or more GCMs [72].

## Results

### Model Results

All four species (*P. fremontii*, *S. gooddingii*, *S. exigua*, and *Tamarix*) produced partial ROC values statistically greater than 1.0 (t-test, p<0.0001), with values ranging from 1.169 to 1.484 (Table S1 for supporting information). Binomial probabilities were p<0.0001, suggesting that all models predicting the distribution of suitable habitat of each species provided significant discriminatory ability as compared with random models (Table S1 for supporting information).

### Environmental Variable Importance and Habitat Association

For all three native species and *Tamarix* temperature variables had the greatest contribution to the models, as quantified by permutation importance, with the largest effect for *P. fremontii, Tamarix* and *S. exigua* (Table 1). The distribution of *S. gooddingii* was influenced by both temperature and precipitation (Table 1).

**Figure 1:**
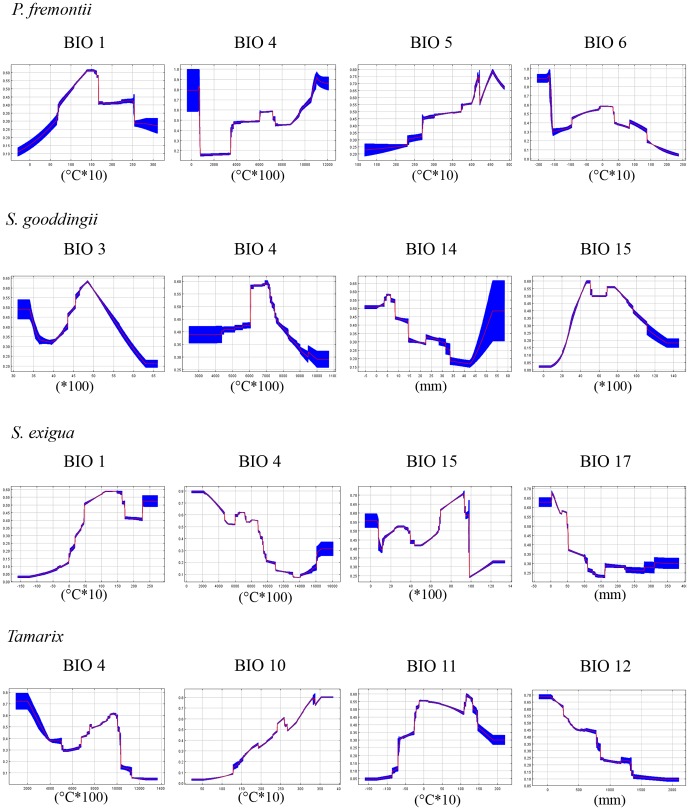
Marginal response curves for each climatic variable. Response curves depicting the probability of suitable habitat related to each climatic variable for *Populus fremontii*, *Salix gooddingii*, *Salix exigua* and *Tamarix*. Units of precipitation variables are measured in millimeters (mm), and temperature variables are degrees Celsius times 10 (°C*10). See Table 1 for full variable names.

The response curves indicated that suitable habitat generally decreased with excess precipitation, and increased with increasing temperature, up to a threshold (Fig. 1). Overall, these results indicate that for the riparian species examined here, temperature variables have a larger influence in determining suitable habitat than precipitation.

### Impact of Climate Change: Range Contraction and Expansion

**Figure 2:**
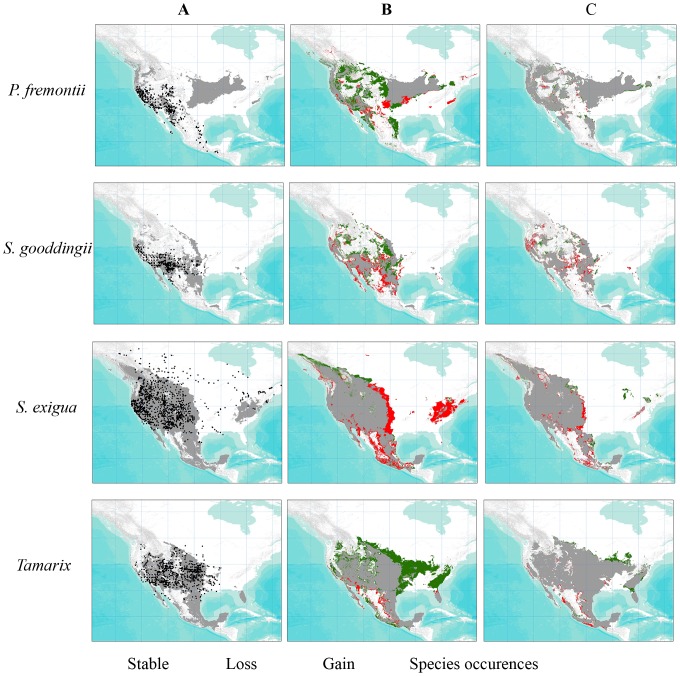
Maxent model outputs showing range expansion and contraction of suitable habitat predicted through time as a result of CC. A) Suitable habitat in current time is shown in grey using a liberal threshold (T10), with the occurrence data overlaid. All occurrence data were downloaded from the Global Biodiversity Information Facility. B-C) Suitable habitat in 2050s and 2080s, predicted by four or more GCMs using a liberal threshold (T10) is also depicted. Areas of loss (red), gain (green), and stable (grey) were calculated by subtracting suitable habitat from the previous time frame.

Under a liberal threshold (T10), four or more GCMs predict that the distribution of suitable habitat for *Tamarix* is expected to shift a total of 71 percent by the end of the century (Fig. 2, Table 2), with a gain of 62 percent and a loss of 9 percent. Of the native species, suitable habitat for *S. exigua* is projected to be the most stable species through time, with only 39 percent of suitable habitat changing (gain of 9 percent and a loss of 30 percent) (Fig. 2, Table 2). While both *P. fremontii* and *S. gooddingii* are expected to gain suitable habitat, over 62 and 38 percent, respectively, up to 40 percent of the suitable habitat for *S. gooddingii* is projected to be lost between now and 2080s (Fig. 2, Table 2). Together, the three native species are expected to have a net gain of nearly 36 percent of current projected suitable habitat by the 2080s (Fig. 2, Table 2).

**Table 2:** Predicted percent change (gain and loss) of projected suitable habitat through time using agreement among four or more GCMs and a liberal threshold (T10).

	Total % Change Current-2080s	Total % Loss Current-2080s	Total % Gain Current-2080s
Species			
*P. fremontii*	80%	17%	62%
*S. exigua*	39%	30%	9%
*S. gooddingii*	77%	40%	38%
*Tamarix*	70%	9%	62%
Native species	60%	24%	36%

### Impact of Climate Change in the presence of Tamarix

**Figure 3:**
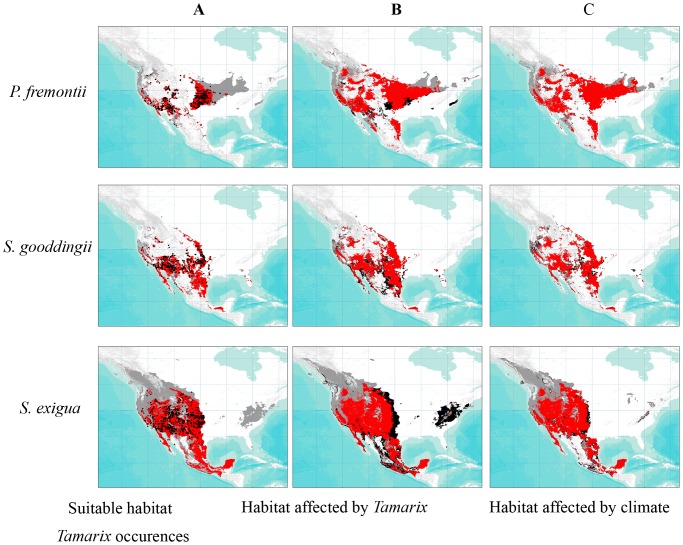
Native species distributions affected by CC or *Tamarix*. Projected distributions of the three native species (grey) showing the range predicted to be affected by either *Tamarix* invasion (red) between current and 2080 (A-C) or CC (black) between 2050 and 2080 (B-C).

We found that nearly 140 percent of future projected native riparian habitat (defined as containing a minimum of one of the three native species modeled here) in western North America, relative to current projected habitat, is expected to have *Tamarix* present by 2080 (Fig. 3, Table 3). *Tamarix* invasion is expected to occur across 200 percent of *P. fremontii* and 174 percent of *S. gooddingii* by 2080 (Table 3). By 2080, over 102 percent of current *S. exigua* habitat is expected to have *Tamarix*, with the effect spread out relatively evenly between 2050 and 2080 (Table 3). When combined with the effect of CC, nearly 156 percent of native habitat is predicted to be affected by either factor by 2080 (Fig. 3, Table 3). *P. fremontii* is projected to experience the greatest impact of climate change and *Tamarix* invasion, with 205 percent of habitat being affected by 2080, followed by *S. gooddingii* (194 percent) and *S. exigua* (116 percent) (Fig. 3, Table 3).

**Table 3:** The number of cells (km^2^) calculated for *P. fremontii*, *S. gooddingii*, *S. exigua*, and all native species together.

	Available habitat	Available habitat	Habitat loss	Habitat Overlap with *Tamarix*	Habitat Loss and *Tamarix*	Available habitat	Habitat loss	Habitat Overlap with *Tamarix*	Habitat Loss and *Tamarix*	Total % impacted by loss	Total % impacted by *Tamarix*	Total % impacted by either factor	Total % impacted by both factors
	current	2050s	2050s	2050s	2050s	2080s	2080s	2080s	2080s	(current-2080s)	(current-2080s)	(current-2080s)	(current-2080s)
	a	b	c	d	e	f	g	h	i	((c+g)/a)*100	((e+h)/a)*100	(((d+e-f)+(g+h-i))/a)*100	((e+i)/a)*100
*P. fremontii*	3688174	5E+06	535414	3572125	380305	5E+06	108988	3809569	83554	17%	200%	205%	13%
*S. exigua*	8806324	7E+06	2072957	4662669	1026756	7E+06	578385	4299096	363179	30%	102%	116%	16%
*S. gooddingii*	2918525	3E+06	736097	2613738	291126	3E+06	424756	2477806	286337	40%	174%	194%	20%
Native habitat	15413023	2E+07	3344468	10848532	1698187	2E+07	1E+06	10586471	148266	29%	139%	156%	12%

Also shown are the percentages of total habitat impacted by; loss of suitable climatic habitat, *Tamarix*, either loss of suitable climatic habitat or *Tamarix*, and both *Tamarix* and loss concurrently.

**Figure 4:**
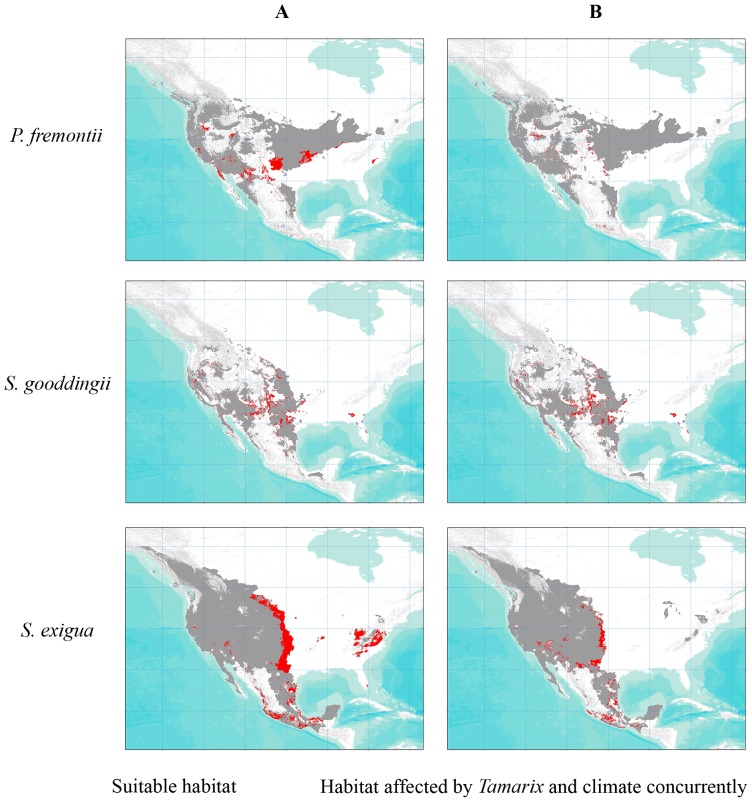
Locations projected to be affected by CC and *Tamarix* concurrently. The projected distributions of three native species depicting the areas with the potential for exotic-by-climate interactions (i.e. the effect of *Tamarix* and habitat loss due to CC) in A) 2050s and B) 2080s.

Climate change and *Tamarix* will also affect native habitat concurrently. We found that 12 percent of current native riparian habitat may be impacted by both CC and the presence of *Tamarix* by 2080 (Fig. 4, Table 3). Nearly 15 percent of *P. fremontii* and *S. exigua* habitat, and 20 percent of *S. gooddingii* habitat is projected to be affected by both factors by 2080 (Fig. 4, Table 3). Contrary to our original hypothesis, concurrent effects were not constrained to the trailing edges of the distribution of native species, although they were more prevalent at the trailing edge for some species, such as *S. exigua* (Fig. 4).

## Discussion

This study demonstrates that CC will likely alter the distribution of three native riparian species in western North America. However, there are two important caveats to address in our study. First, despite statistically significant pROC scores (Table S1 for supporting information), the models developed for *P. fremontii* and *S. exigua* showed a relatively high false negative rate (Fig. 1). This is likely a result of over-fitting in the Maxent algorithm [73], and thus projected changes in both *P. fremontii* and *S. exigua* distributions may be especially conservative. Additionally, all future projections for the species modeled here are based upon only five GCMs and one moderate climate change scenario (SRES A1B). Although our rationale for using the five GCMs chosen is based upon previous research of climatic conditions in the southwestern U.S. [58], we did use a moderate emissions scenario, which is conservative in light of current CO_2_ emission rates [74]. Thus, model results presented here are likely conservative estimates of habitat turnover, and actual future climatic conditions may deviate from those used here.

### Vulnerability of Native Species to Climate Change

In contrast to our original hypothesis that climate change would have the greatest impact on *P. fremontii*, followed by *S. gooddingii* and *S. exigua*, we found that *S. gooddingii* is projected to experience the greatest loss of suitable habitat due to CC, followed by *S. exigua* and *P. fremontii* (Table 2). These results are in partial agreement to those reported by Grady *et al*. [39], who experimentally demonstrated greater sensitivity in transfer climate distance of *P. fremontii* and *S. gooddingii* relative to *S. exigua*. In our study, the greatest decline in habitat was predicted in the next thirty years, with moderate declines between 2050 and 2080 (Fig. 2). Although it is unclear why the next thirty years would show greater changes than the following 30 years, it may be that a threshold is reached in the first 30 years that triggers major changes. Following a similar pattern with many other species [1], [75], [76], suitable habitat of the three native species is projected to decline along the trailing edges of the species range with little to moderate increases in habitat at the leading edges (Fig. 2).

Two patterns became apparent when examining the bioclimatic variables which correlated with the distribution of these native riparian species. First, variables related to temperature had the greatest permutation importance on the model building process for *P. fremontii*, and *S. exigua* (Table 1), and for both native species increases in temperature correlated with increased habitat suitability (Fig. 1), with some thresholds present. Second, both precipitation and temperature were important for *S. gooddingii*, with suitable habitat generally increasing with temperature up to a threshold (Table 1). However, increases in precipitation led to a decrease in suitable habitat (Fig. 1). Together, these patterns suggest that while variable contribution differs between the native species, drier, warm climates provide more suitable habitat. While this may be surprising, a number of other studies have documented that water use of riparian species is largely influenced by the availability of groundwater rather than rainfall [77]–[79].

### Tamarix and Climate Change

Current estimates indicate that *Tamarix* occupies an area of 356,241 hectares in the western United States [80]. Our results indicate that the climate niche of *Tamarix* (Fig. 1) is approximately 300 times greater than the realized niche [81], suggesting that there is extensive climatically suitable habitat that this species may yet invade. Additionally, there is substantial overlap in projections of suitable habitat between results generated here and those reported by other studies. Jarnevich *et al*. [37] used 29 climatic, topographic, and geographic variables, including distance to water, to characterize the distribution of *Tamarix*, and found present potential habitat throughout the western US, with distinct patches occurring in Utah and Arizona, along the Pacific crest, regions of Montana and Idaho, and along river systems east of the Rocky Mountain Range. Morisette *et al*. [38] used remote sensing data to examine the current realized niche, and similar to our results, found significant concentration of suitable habitat in the southwestern US, but predicted greater occurrence especially in Texas. The *Tamarix* suitability model developed here predicts suitable habitat in almost all of these regions, despite our use of only five bioclimatic variables, indicating that bioclimatic variables alone can accurately characterize a riparian species niche over broad geographic scales.

The bioclimatic variables describing the niche of *Tamarix* occurrences (Table 1) were consistent with those reported by other studies. We found probability of suitable habitat to increase with mean temperature of the warmest quarter, while the remaining bioclimatic variables concerning precipitation had little influence on habitat suitability (Fig. 1). Jarnevich *et al*. [37] also found mean temperature of the warmest quarter to be a significant predictor of suitable habitat while precipitation variables contributed less than 10 percent.

In support of our original hypothesis, we found that *Tamarix* invasion of riparian habitat is expected to increase with CC (Fig. 2, Table 2). Suitable habitat of *Tamarix* is projected to gain approximately 62 percent of the current suitable habitat by 2080 (Table 2). This finding is contrary to that reported by Bradley *et al*. [24], who predicted *Tamarix* distribution to remain relatively stable through time. The discrepancy is likely due to the fact that Bradley *et al*. [24] used a different modeling algorithm, Mahalanobis distance [82], [83], versus the Maxent algorithm used here. Although no studies directly compare the two modeling approaches, there is evidence to suggest that Maxent performs better than Mahalanobis distance [84]. Some of the habitat gain of *Tamarix* predicted here is projected to occur at latitudes higher than where the species is currently found (Fig. 2). Gaskin and Kazmer [85] found *T. chinensis* and *T. ramosissima* introgression to follow strong latitudinal clines, with a higher occurrence of *T. chinensis* alleles at the southern edge and *T. ramosissima* alleles at the northern edge. This pattern is likely driven by greater frost tolerance in *T. ramosissima* than *T. chinensis* [86]. While projected increases in latitude of *Tamarix* (Fig. 2) should increase the prevalence of *T. ramosissima* alleles, warmer climates could decrease the selection pressure for frost tolerance, resulting in *T. chinensis* alleles becoming more widespread.

### Implications for Management and Conservation Efforts

In the context of restoration and conservation efforts, our results highlight three main conclusions. First, mitigation efforts should focus on areas where native habitat is projected to be affected by CC and the presence of *Tamarix* (Fig. 4). We found that 20 percent of the distribution of *S. gooddingii* is projected to be affected by both habitat loss and *Tamarix*, followed by 16 percent of *S. exigua* habitat, and 13 percent of *P. fremontii* habitat (Table 3). Given the projected future anthropogenic impacts on riparian habitat (reviewed in [23]), even relatively small projected effects can have disproportionate impacts. This is especially true when the habitat in question is rare- largely due to anthropogenic factors (e.g., cattle grazing, water diversions, farming, dams) [16]. With such rare habitat types, even relatively low mortality may result in bottlenecks and local extinction. For example, in a survey by Gitlin *et al*. [10], less than two percent of the riparian habitat surveyed had *P. fremontii*, but *P. fremontii* experienced greater than 15 percent mortality. Because riparian areas are predicted to be most susceptible to extirpation of native species and endemic species [87], *Tamarix* removal and restored stream flows, which may alleviate loss from predicted CC and increase native species regeneration [88], [51], should be a priority.

Second, rehabilitation of *Tamarix* altered habitat, involving replanting of native riparian species, should begin in areas where *Tamarix* is expected to decline as a result of CC as reflected in Figure 2. Based on the *Tamarix* response function generated here (Fig. 1), we can expect that *Tamarix* will decrease in regions projected to become wetter. Thus, re-vegetation efforts with *Populus* and *Salix* species may be particularly successful [89], [90], especially when planted in proportions which maximize associated community diversity [91].

Lastly, genetics-based approaches offer a method to restore habitat in suboptimal areas (e.g., in regions with a high likelihood of excess temperatures, or where *Tamarix* has been present). Planting with source populations known to exhibit increased tolerance to drought and/or higher temperatures, as established through field trials, can increase survival rate and performance [39]. Similarly, managed relocation, based on genetic pre-adaptation, can also be utilized to mitigate the effects of riparian exotics by identifying populations that can better compete with *Tamarix*, as has been shown with other native species and their competing invasives. For example, Goergen *et al*. [92] showed that when native grass species (*Poa secunda* and *Elymus multisetus*) from different source populations were grown with cheatgrass (*Bromus tectorum*), native grasses from source populations of invaded sites better tolerated competition with cheatgrass than native grasses from uninvaded sites. We can take genetics approaches one step further by identifying source populations and genotypes of native species that can simultaneously cope with both CC and invasive species. Such plants may be identified in regions where *Tamarix* occurrences overlap with native species distributions and CC has already resulted in near extirpation of native species [areas with both *Tamarix* occurrences (Fig. 3A) and depicted in black (Fig. 3B)], leaving only genotypes that have persisted in the presence of both *Tamarix* and climate change.

### Future Directions: Incorporating Indirect Effects of *Tamarix* and Climate Change on Native Habitat

Although others have reviewed indirect effects of anthropogenic CC on riparian systems in the context of stream flow and human activity (see [23]), there are at least three reasons why *Tamarix* may exert even greater influence on riparian habitats than we predict due to the effects of multiple stressors, community composition and intraspecific variation on the productivity and diversity of native riparian habitat. Thus, our findings may be conservative in light of possible indirect ecological effects between native riparian species, climate and *Tamarix* in the context of CC as follows.

First, *Tamarix* can negatively impact riparian trees already experiencing other stressors. The presence of *Tamarix* seedlings has been shown to be unrelated to *Populus* and *Salix* growth or survival in benign environments [88]; however, *Tamarix* is most abundant along altered streams and rivers where *Populus* recruitment is the lowest [19]. Additionally, Gitlin *et al*. [10] examined the relationship between *Tamarix* density and *P. fremontii* mortality during a severe drought. As *Tamarix* density rose, there was a proportional increase in the mortality of *P. fremontii* (r^2^ = 0.69) that reached 97 percent when the density of *Tamarix* was approximately 65 percent. Since this mortality relationship was manifested during a record drought, it argues that when *Tamarix* is present, the addition of additional stressor can detrimentally impact a native riparian species. Thus, under CC, we would expect an increase in *Tamarix* due to both an expansion of suitable climatic habitat (as reported here) and projections of escalating human impacts to river systems (reviewed in [23]), and a corresponding rise in mortality of native riparian species due to the presence of *Tamarix* and climate related stress. The combination of these circumstances could act synergistically on *Tamarix* abundance.

Second, *Tamarix* can indirectly impact native riparian habitat by altering the associated community, which can negatively affect both the diversity and productivity of these ecosystems. In *Tamarix* dominated stands, community composition of invertebrates and overall diversity of both invertebrates [93], [94] and birds [95] have been recorded as less than in habitat dominated by native riparian species. Negative effects on biodiversity were also discovered in a stream where aquatic macroinvertebrates were less on *Tamarix* than cottonwood leaf litter [96]. The presence of the tamarisk beetle (*Diorhabda* spp.), which was first released in 2001 as a biocontrol agent for *Tamarix* can also lead to different community assemblages [97]. Furthermore, impacts on the community can occur belowground. Meinhart & Gehring [35] showed that the presence of *Tamarix* altered the arbuscular (AMF) and ectomycorrhizal fungal (EMF) communities of *P. fremontii*, decreasing the colonization rate by half. Since mycorrhizal fungal associations can positively impact the growth of native species [98], and biodiversity promotes ecosystem stability [99], accounting for their presence and structure can further inform how these riparian species and their associated communities might respond to CC.

Lastly, many studies have demonstrated that populations of foundation trees are locally adapted, and intraspecific responses to climate change may be highly variable [77], [100], [101]. Accounting for these population level differences can impact predictions of how species respond to CC. Oney *et al*. [102] compared model results from an ENM (Maxent) and a population-based model (Universal Transfer Functions) for *Pinus contorta* and found that intraspecific genetic variation buffered the species from CC, indicating that ENMs may be under-predicting future suitable habitat. Furthermore, intraspecific genetic variation in productivity will impact associated community members, leading to predictable changes in biodiversity as a result of CC [103]. Although complex, merging the interactions between the indirect effects discussed here and the direct effects reported through our modeling efforts should provide better management strategies to mitigate the effects of both climate change and invasive species on native species and the rich communities they support.

## Conclusions

This study is a first step toward integrating multiple environmental challenges (CC and invasive species) to inform conservation and management decisions. Results presented here can help prioritize areas for conservation and restoration by identifying regions where 1) invasive *Tamarix* and CC will jointly impact riparian species in the future, 2) *Tamarix* is expected to increase as a result of climate change, and 3) where the unique evolutionary history of native species is likely to have selected for genotypes that possess high drought tolerance and/or are highly competitive with *Tamarix*. Identifying these superior genotypes may be especially important to utilize in future mitigation efforts. Through provenance trials, we can experimentally identify source populations for use in restoration that are best adapted to future climate, exotics, or climate-by-exotic interactions (e.g., [39], [40]). Together, this information should increase the success of conservation and restoration efforts in the face of multiple stressors.

## Supporting Information

Table S1 **Partial ROC scores for each of the four species modeled, followed by the standard deviation.** An (*) indicates significance at p<0.0001 using a t-test.
(DOCX)
