# Supplementary material for: Incorporating Climate Change and Exotic Species into Forecasts of Riparian Forest Distribution
Source: PLoS One. 2014 Sep 12;9(9):e107037. doi: 10.1371/journal.pone.0107037 (PMC4162564; doi:10.1371/journal.pone.0107037)
Supplement: Table S1 — Partial ROC scores for each of the four species modeled, followed by the standard deviation. An (*) indicates significance at p<0.0001 using a t-test. (DOCX) [file pone.0107037.s001.docx]

| Species | partial ROC | ± S.D. |
| --- | --- | --- |
| *P. fremontii* | 1.169* | 0.026 |
| *S. exigua* | 1.331* | 0.054 |
| *S. gooddingii* | 1.304* | 0.016 |
| *Tamarix* | 1.484* | 0.021 |
